# Classic and New Diagnostic Approaches to Childhood Tuberculosis

**DOI:** 10.1155/2012/818219

**Published:** 2012-03-12

**Authors:** Gladys Guadalupe López Ávalos, Ernesto Prado Montes de Oca

**Affiliations:** ^1^Interinstitutional Posgrade in Science and Technology, Research Center in Technology and Design Assistance of Jalisco State (CIATEJ, AC), National Council of Science and Technology (CONACYT), 44270 Guadalajara, JAL, Mexico; ^2^Molecular Biology Laboratory, Biosecurity Area, Pharmaceutical and Medical Biotechnology Unit, Research Center in Technology and Design Assistance of Jalisco State (CIATEJ, AC), National Council of Science and Technology (CONACYT), 44270 Guadalajara, JAL, Mexico

## Abstract

Tuberculosis in childhood differs from the adult clinical form and even has been suggested that it is a different disease due to its differential signs. However, prevention, diagnostics, and therapeutic efforts have been biased toward adult clinical care. Sensibility and specificity of new diagnostic approaches as GeneXpert, electronic nose (E-nose), infrared spectroscopy, accelerated mycobacterial growth induced by magnetism, and flow lateral devices in children populations are needed. Adequate and timely assessment of tuberculosis infection in childhood could diminish epidemiological burden because underdiagnosed pediatric patients can evolve to an active state and have the potential to disseminate the etiological agent *Mycobacterium tuberculosis*, notably increasing this worldwide public health problem.

## 1. Introduction

Tuberculosis is the leading cause of death worldwide, with over 1.5 million deaths per year. This disease is caused by *Mycobacterium tuberculosis*, which is an acid-fast bacilli, and it is transmitted mainly by the airway [1]. While adult TB cases are often easily recognizable, due to typical symptoms (radiological features and a positive sputum smear), TB in childhood is frequently more difficult to diagnose due to the atypical radiological features and the difficulty to expectorate [2]. Furthermore, there is a significant morbidity and mortality in children worldwide [3], with a majority of cases of latent TB infection (LTBI) and active disease occurring in developing countries [4]. Childhood tuberculosis is commonly extrapulmonary, disseminated, and severe, especially in children under 3 years of age, and it is associated with high morbidity and mortality [5]. Approximately, 15–20% of all TB cases in sub-Saharan Africa are in children [6]. 

The natural history of TB in children and pediatric patients follows a series of steps. Phase 1 occurred 3–8 weeks after primary infection. This is the end of incubation period and the initiation of well-defined signs: fever, erythema nodosum, a positive tuberculin skin test response, and formation of the primary complex visible on chest radiography. Phase 2 occurred 1–3 months after the phase 1. In this period, the bacillus can migrate to other parts of the body via the blood and represented the period of the highest risk for the development of tuberculous meningitis and miliary tuberculosis in young children. This is the phase where dissemination of the bacillus most frequently occurs. Phase 3 occurred 3–7 months after primary infection. This is the period of pleural effusions in >5 years old children and bronchial disease in <5 years old children. Phase 4 presents after 1–3 years of the phase 1. In this period, the osteoarticular tuberculosis in children with 5 years or less appears. Phase 5 occurs up to 3 years after phase 1 and it is presented after calcification was completed. Until this phase, manifestations of classical adult tuberculosis appear [7, 8].

From a tuberculosis control point of view, programs place an almost exclusive emphasis on adults with sputum smear positive. Tuberculosis in children remains a neglected area of research despite considerable morbidity and mortality [9–11]. The common perception is that children rarely develop severe forms of tuberculosis and contribute little to the maintenance of the tuberculosis epidemic [12]. Nevertheless, children can transmit *Mycobacterium tuberculosis*, this contention is documented in a lot of reports and community outbreaks. Controversy exists regarding this fact [13, 14]. Indeed, children do contribute a substantial proportion of the global tuberculosis disease burden [12], however, most programs for TB control are limited because they target and treat only active cases [15]. Most TB cases in children present as latent tuberculosis (LTBI) and active disease occurring mainly in developing countries [4]. Without treatment, the majority of infants aged under 1 year dies due to TB. Even with effective antimicrobial therapy, severe TB continues to occur in young children [16].

One interesting issue about the disease in children is that age determines the progress to disease; infants are at the highest risk than older children, so it is possible to categorize all children with 3 years or less in high risk [17]. Other factors can be HIV, malnutrition, measles, and *Bordetella pertussis *infection [15]. The aim of the present review is to summarize classic and new approaches for the diagnosis of childhood TB and the challenges that this goal represents.

## 2. Epidemiology and Burden of Disease

The risk of infection depends on the duration of exposure, the closeness of contact, and the microbial load of the source case [15, 18]. From 1987 to 1991, the number of TB cases among children less than 5 years old in the United States increased by 49% from 674 to 1006 cases. According to the World Health Organization (WHO) estimations, in 1990, there were 8 million new cases of TB and 3 million deaths due to the disease worldwide; 13 million new cases and 450,000 deaths were among children less than 15 years old [7]. In 1994, it was estimated that the global incidence of tuberculosis (TB) in children aged 0–14 years would be more than 1 million cases by 2000, with half of these cases occurring in Africa. This represents a worldwide increase of 36% from the 1990 estimate [15]. From the estimated 8.3 million new TB cases diagnosed in 2000, 884,019 cases (11%) were children, and their contribution to the disease burden in endemic areas is estimated to be even higher [4, 17, 19]. Notably, the highest case burden, an estimated 95% of all TB cases, is found in developing countries [20]. Regional data from the World Health Organization (WHO) in 2007 showed that smear-positive TB in children aged <14 years accounted for 0.6–3.6% of reported cases. However, because ~95% of cases in children <12 years of age are smear negative, these data underestimate the true burden of TB. In 2010, of the ~1 million estimated cases of TB in children worldwide, 75% occur in the 22 highest-burden countries [21]. In a report from a tuberculosis endemic area in Cape Town, South Africa, children less than 13 years old contributed 13.7% of the total disease burden and experienced a tuberculosis incidence rate in excess of 400/100,000 per year [17]. Although childhood tuberculosis contributes to only 3–6% of the total caseload in industrialized countries, it makes up a large proportion (15–20%) of all TB cases in developing countries [4], moreover, infected children are a reservoir from which many adult cases will arise [7].

In developing countries, the risk for TB infection and disease is relatively uniform in the population; annual rates of infection often exceed 2% depending mainly upon exposition to infected persons [7]. More recent global and country-specific estimates do not stratify these estimates by age, in part because of the inherent difficulties with confirming the diagnosis of childhood TB [14, 22]. Unfortunately, even as the TB reports in adults are very common, no recent epidemiological reports have been published on the global TB burden in children, this is due to the lack of adequate diagnostic methods with high precision and sensibility and the low priority given to children in global TB control programs [23].

## 3. Diagnostic Difficulties in Childhood Tuberculosis

The diagnosis of tuberculosis in children is complicated mainly because (i) TB can mimic many common childhood diseases, including pneumonia, generalized bacterial and viral infections, malnutrition, and HIV [18]; (ii) the absence of a practical reference test or gold standard [12, 24]; (iii) the inability of preadolescent patients to expectorate sputum [14, 22]; (iv) the nonspecific clinical presentation [9, 11, 19, 22]; (v) the lower bacillary load often smear negative [3, 14, 19, 22, 25–27]; (vi) confirmation by culture of *Mycobacterium tuberculosis*, the gold standard of diagnosis in adult TB, rarely exceeds 30–40% sensitivity [3] (although it may be considerably higher in children with advanced disease) [19] even when using gastric aspirates, induced sputum, liquid media, and polymerase chain reaction (PCR) [6, 14, 22] and, in addition, the distinction between recent primary infection and active disease is highly problematic [28] even the gastric aspirates continue to be the best specimens for testing for suspected pulmonary TB in children [29] with 30–40% sensitivity [19]. Symptoms of childhood TB are nonspecific, and up to 50% of children may be asymptomatic in early stages of the disease. Other aspects like HIV infection and malnutrition make difficult the diagnosis of childhood tuberculosis [20] (Table 1).

Most children with TB are classified as smear-negative pulmonary TB (PTB) for the reasons mentioned above (difficulty to expectorate, lack of equipment to gastric lavage, etc.) which is an inappropriate term as a smear or culture has not usually been done. This leads to difficulties in determining the true extent of PTB in children in different areas and circumstances. Extrapulmonary TB (EPTB) accounts for up to 20–30% of the total caseload of TB in children, and the diagnosis is usually easier than PTB because of the characteristic clinical features like lymphadenopathy with or without scrofula, spinal deformity, disseminated disease, meningitis, effusions (pleural or pericardial), or painless ascite [15]. The isolation of *Mycobacterium tuberculosis *takes several weeks. Consequently, the diagnosis of TB in children is often supported only by epidemiological, clinical, and radiographic findings in the presence of a positive tuberculin skin test [29].

The value of the classic diagnostic is named: (1) exposure to an adult index case; (2) chronic respiratory symptoms that do not respond to broad-spectrum antibiotics; (3) documented weight loss or failure to thrive; (4) a positive tuberculin skin test (TST); (5) the presence of suggestive signs on the chest radiograph (CXR), which is greatly reduced in endemic areas where exposure to and/or infection with *Mycobacterium tuberculosis *is common [12, 14, 15, 21, 22, 24, 30]. These criteria are less helpful in endemic areas where a positive TST result is common and exposure to *M. tuberculosis *is often undocumented [19] (Tables 1 and 2). For all these reasons, many children with TB are never diagnosed or registered as cases of TB [14, 22]. Furthermore, the consequences of missed diagnosis in children are severe, as untreated children have a high probability of developing active TB, usually within two years of infection [31, 32] (Table 2).

The difficulty to obtain samples for TB diagnosis in children has led researchers to create smart approaches as “*la cuerda dulce*” (sweet string), reported by Chow et al. in 2006. They provide a technique which consists of a coiled nylon string inside a gel capsule. The string unravels through a hole in the end of the weighted capsule as it descends into the stomach and the capsule then dissolves in it, allowing the string to become coated with gastrointestinal secretions containing whatever pathogens are present. A few hours later (~4), the capsule passed in the feces. This methodology is well tolerated by children and is less invasive than the gastrointestinal lavage [32]. 

## 4. Classical Diagnosis

### 4.1. Clinical Symptoms Approach

The use of well-defined symptoms improves diagnostic accuracy of PTB [24]. With clinical symptoms approach only, the status can be classified in two, suspected TB or probable TB. Two situations lead the clinician to suspect that a child has tuberculosis. The first is (a) history of chronic illness with clear symptoms: cough and/or fever, weight loss or failure to thrive, an inability to return to normal health after measles or whooping cough, fatigue, and wheezing; second, (b) one or more of the following: malnutrition, lymphadenopathy, chest signs, hepatomegaly and/or splenomegaly, meningeal signs, and/or ascites. For probable TB, in addition to suspected TB, the child presents TST positive, suggestive chest radiological appearances as pleural effusion, caseation of biopsy material, poor response to 2 weeks antibiotic treatment, and/or favourable response to antituberculous treatment (weight gain and loss of signs) [19, 33]. In pediatric TB, the most common symptoms are pulmonary parenchymal disease and intrathoracic adenopathy accounting for 60–80% of all cases. Among extrapulmonary manifestations, lymphadenopathy is the most common (67%), followed by central nervous system involvement (13%), pleural (6%), miliary and/or disseminated (5%), and skeletal (4%) TB form. Disseminated (miliary) disease and TB meningitis are usually found in very young children (<3 years-old) and/or HIV-infected children [21]. TB meningitis occurs when the child has contact with a suspected or confirmed case. About 50% of childhood TB cases are discovered in this manner [7].

In general, there is a sense of skepticism regarding the potential diagnostic value of symptom-based approaches; however, the natural history of childhood tuberculosis demonstrates that symptoms may have diagnostic value if appropriate risk stratification is applied. Marais et al. in 2006, conducted a study to assess the ability to diagnose TB in HIV-negative children with symptoms and concluded that PTB can be diagnosed with a reasonable degree of accuracy in HIV-uninfected children (with a high degree of accuracy in the low-risk group), using a simple symptom-based approach. This offers the exciting prospect of improving access to antituberculosis treatment for children in resource-limited settings [12].

Houwert et al. in 1998, conducted a prospective evaluation of the WHO criteria to see if it was specific and predicted the situation of TB in children and they conclude that the diagnosis of tuberculosis must be more seriously considered when a child presents the triad of the above mentioned criteria [30]. Marais et al. 2005, evaluated whether well-defined symptoms have a diagnosis value in children and a standard symptom-based questionnaire was completed and reported symptoms were individually characterized. A tuberculin skin test (TST) and chest radiograph (CXR) were performed in all children. In this study, well-defined symptoms had excellent diagnostic value [34].

### 4.2. Radiologic Studies

The radiography became available after the First World War, and since that time, PTB detection became easier [8]. Evidence of pulmonary TB in chest radiographs varies, but usually radiographs show enlargement of hilar, mediastinal, or subcarinal lymph nodes and lung parenchymal changes, hilar lymphadenopathy with or without a focal parenchymal lesion. The most common findings are segmental hyperinflation then atelectasis, alveolar consolidation, interstitial densities, pleural effusion, and, rarely, a focal mass. Cavitation is rare in young children but is more common in adolescents, who may develop reactivation disease similar to that seen in adults [7, 23]. High-resolution computed tomography is the most sensitive tool currently available to detect hilar adenopathy and/or early cavitation [19] (Table 2).

### 4.3. Algorithms

There are point-scoring systems to make a diagnostics classifications. Diagnostic algorithms were developed to deal with these diagnostic difficulties and provide the health care worker with a rational, stepwise tool to identify children in need of TB treatment. They are very helpful and very easy to use in countries with restricted technology, but a few of them are used now [6].

### 4.4. Mycobacterial Detection and Isolation

Microbiological confirmation of TB in young children is not routinely attempted in many high burden settings due to the difficulty in obtaining samples and the poor performance of smear microscopy [2]. Diagnosis of TB still relies primarily on examination of Acid-Fast Bacilli- (AFB-) stained smears from clinical specimens in adults, however, children with pulmonary TB usually do not cough up voluntarily, either because they do not produce sputum or because it produces discomfort. When sputum samples cannot be obtained, gastric aspirate samples are used for detection and isolation of *M. tuberculosis* [35]. Most of the current TB diagnostic methods were developed over a century ago. In 1898, Neunier became the first person to culture stomach contents for the evidence of tuberculosis in children [23, 36], so even with this method, fewer than 20% of children with TB have a positive AFB smear of sputum or gastric aspirate. For many years, the collection of three consecutive early morning gastric lavages or gastric aspirate samples has been the accepted method for attempting microbiological confirmation even as the yield is very low and that in many populations cannot be performed due to the lack of infrastructure. In addition low pH is well known that kills tuberculous bacilli, indicating that stomach pH may inhibit TB survival for subsequent culture [23]. More recently, a number of less invasive alternative methods have been proposed, including induced sputum (administration of an inhaled bronchodilator followed by nebulized hypertonic 3–5% saline and then nasopharyngeal aspiration or expectoration of mucus from lower respiratory tract), in the nasopharyngeal aspiration, a cannula elicits a cough reflex and the sweet string test mentioned above [2].

All of these alternative ways of sampling have been made to increase yield because a positive culture is regarded as the “gold standard test” to establish a definitive diagnosis of TB in a symptomatic child [19]. If culture is negative, diagnosis is made on the basis of a positive TST, clinical and radiographic findings suggestive of TB, and history of contact with an adult source case the child may be diagnosed with positive TB based on symptomatology. This measure was taken because the yields in child are less than 50%. Zar et al. investigate whether sputum induction can be successfully performed in infants and young children with and without HIV and determine the utility of salbutamol-induced sputum compared to gastric lavage (GL) for the diagnosis of pulmonary tuberculosis. They conclude that sputum induction can be effectively performed and is well tolerated and safe even in infants and this induction is better than GL for the isolation of *M. tuberculosis *in both HIV-infected and uninfected infants and children [35].

### 4.5. Smear Microscopy

Advances have been done in the performance of smear microscopy for the rapid detection of MTB, for example, the concentration of specimens by centrifugation or the change of the staining of carbol fuchsin (Ziehl-Neelsen or Kinyoun) for a fluorescent dyes (auramine-rhodamine), which both increases sensitivity and reduces the time for screening [37]. However, even under optimal circumstances, the sensitivity of smear microscopy for the diagnosis of childhood TB remains less than 15%, except in older children with adult-like disease [2].

### 4.6. Tuberculin Skin Test (TST)

The Tuberculin skin test, or Mantoux TST, is based on the detection of a cutaneous delayed-type hypersensitivity response to purified protein derivative, a poorly defined mixture of antigens present in *M. tuberculosis, Mycobacterium bovis *Bacille Calmette-Guerin (BCG) and several nontuberculous mycobacteria [38]. TST is the standard method for detecting infection by *M. tuberculosis*. The reaction is measured as millimeters of induration after 48 to 72 hours [7].This test was the only method available for the diagnosis of latent tuberculosis infection (LTBI) until very recently.

With a TST, it is not possible to assert or deny the presence of TB, but it only indicates infection with a mycobacterium. In a child who has not been BCG-vaccinated, a TST has been defined as positive when the diameter of skin induration is >10 mm, and in a BCG-vaccinated child, when the diameter of induration is >15 mm. A negative TST does not exclude TB [7] and some induration (5–14 mm) could be supportive if the clinical features and contact history are suggestive [15]. Furthermore, the utility of this conventional test is hampered by technical and logistical problems: potential for false-positive and false-negative results; problems in administration and interpretation; difficulty in separating true infection from the effects of prior BCG vaccination, infection due to nontuberculous mycobacteria [4, 26, 39]. In children with debilitating or immunosuppressive illnesses, malnutrition, or viral (as HIV) and certain bacterial infections, the yield is unknown, but it is certainly higher than 10%. Moreover, false-positive reactions to TST are often attributed to asymptomatic infection by environmental nontuberculous mycobacteria [7, 38].

### 4.7. Polymerase Chain Reaction (PCR)

Diagnostic PCR is a technique of *in vitro* DNA amplification that uses specific DNA sequences (oligonucleotides) as effective fishhooks for the DNA/cDNA of microorganisms. In theory, this technique can detect a single organism in a lot of specimens such as sputum, gastric aspirate, pleural fluid, cerebrospinal fluid, blood, and urine. Various PCR assays, most using the mycobacterial insertion element IS6110 as the DNA marker for *M. tuberculosis*-complex organisms, have a sensitivity and specificity greater than 90% for detecting pulmonary TB in adults [7] (Table 3).

This is a rapid, sensitive, specific, and reasonable-cost [11] method for the detection of *M. tuberculosis *in clinical samples. The PCR may be used to (a) diagnose tuberculosis in difficult samples with negative microscopic examination, negative culture, or with scarce sample; (b) determine if the organisms in the sample are *M. tuberculosis *or atypical mycobacteria; (c) identify the presence of genetic variations like a mutations or deletions known to be associated with resistance to some antimycobacterial agents [27].

Studies in children have obtained better sensitivity by PCR than by culture. In 2001, Gomez-Pastrana et al. [29] reported a comparison between sensitivity of culture and PCR showing higher sensitivity for the latter. PCR may have a special role in the diagnosis of extrapulmonary TB and pulmonary TB in children since sputum smears are usually unrevealing in these cases [7]. However, these tests are not performed correctly in all clinical laboratories. The cost involved, the need for sophisticated equipment, the limitations in their specificity, the need to obtain multiple samples to optimize yield and scrupulous technique to avoid cross-contamination of specimens preclude the use of PCR techniques in many developing countries [11].

Montenegro et al. in 2003, reported a heminested PCR assay which specificity was 67%. This was significantly higher than Löwenstein-Jensen culture (54%) or AFB stain (42%) for children with highly probable tuberculosis. PCR detection rates for culture-positive specimens were 100% for smear-positive samples and 76.7% for smear-negative samples. The specificity of PCR was 100% in control children. Compared with culture, PCR showed a sensitivity of 90.4%, a positive predictive value of 89%, a specificity of 94%, and a negative predictive value of 95% [11]. In another research in pulmonary TB, the sensitivity ranges from 4–80% and the specificity was 80–100% [27]. In tubercular mediastinal adenopathy, Gomez-Pastrana et al. [29] conducted a prospective study comparing nested PCR, mycobacterial cultures, and the clinical diagnosis to investigate which test had the higher sensitivity and specificity. The sensitivity of PCR of gastric lavage/bronchoalveolar lavage was 56.8% in children with clinically active disease. Authors conclude that nested PCR is a rapid and sensitive method for the early diagnosis of TB in children. Additionally, other unique sequences of *M. tuberculosis* have been suggested as diagnostic test for TB, because they are absent in *M. africanum, M. microti, M. bovis*, and* M. bovis BCG *[43].

### 4.8. In-House Nucleic Acid Amplification (NAA) Assays

These assays are highly dependent of operator's skills. Performance is also influenced by the choice of target sequence and DNA extraction method. Interpretation of the performance of these assays in pediatric TB suspects is confounded by the lack of a sensitive and specific reference standard. When compared with culture, the sensitivity of NAA for the diagnosis of childhood TB is typically low (40–83%). However, it appears, at least from some reports, that NAA identify a group of children who are clinically diagnosed with TB but in whom mycobacterial culture is negative. This means that with a proper technique it could be done efficiently [2].

### 4.9. Adenosine Deaminase

Considering the low yield of smear and culture in PPTB, nonmicrobiological methods may provide new tools for diagnosis. Adult studies have shown increased levels of adenosine deaminase (ADA) in pleural TB and TB-caused meningitis, both paucibacillary forms of TB, and have advocated its use in diagnosis. Due to this evidence, a serum ADA has already been evaluated in a childhood population with a very high sensitivity (100%) and specificity (90.7%) for pulmonary TB. This study demonstrated the great potential of this technique because it has significant difference in serum ADA levels between children with disease and infection. However, there were several weaknesses in the study design, including unclear case definition, exclusion of nontuberculous patients, and a relatively small TB patient population (20 with active disease) [23].

In the case of extrapulmonary TB, ADA measurement can be helpful, but its sensitivity and specificity varies widely and has been lower than multiplex PCR using primers for IS6110, dnaJ, and hsp65 [44]. Specifically, a meta-analysis of 63 studies of ADA in tuberculous pleuritis reveals that the sensitivity of the test is of 0.92 (95% CI 0.90–0.93) and specificity of 0.90 (95% CI 0.89–0.91) [45] (Table 3).

### 4.10. Serology and Antigen Detection

In absence of good diagnostic method for tuberculosis, the interest in serodiagnosis has been increased [27]. Serological tests vary in a number of features, including antigen composition (38 kDa, Ag 60, and lipoarabinomannan, LAM), antigen source (native or recombinant), chemical composition (protein or lipid), extent of antigen(s) purification, and immunoglobulin detected. The majority is based on the enzyme-linked immunosorbent assay (ELISA) rapid versions use various immunochromatographic formats, with lateral flow being the most popular [31].

Hussey et al. in 1991 [46], used an autoclaved suspension of *M. tuberculosis *to detect antibodies in serum from 132 children with clinical pulmonary TB; the test was 62% sensitive and 98% specific. Imaz et al. in 2001 [25] reported the usefulness of the recombinant 16-kDa antigen (re-Ag16) of *Mycobacterium tuberculosis *in the serodiagnosis of tuberculosis (TB) in children measuring the values of IgA, IgM, and IgG and an increased mean antibody response to reAg16 was observed in contact children compared with nonmycobacterial disease patient with a 95% of specificity. A combining result of the IgG and IgA assays led to 43% positivity in children with active TB. Mycobacterial antigen detection has been evaluated in adults, but rarely in children [7].

Serology has found little place in the routine diagnosis of tuberculosis in children, even though it is rapid and does not require specimen from the site of disease. Sensitivity and specificity depend on the antigen used, gold standard for the diagnosis of tuberculosis, and the type of tubercular infection. Though most of these tests have high specificity, their sensitivity is poor because several factors can alter the results such as age, exposure to other mycobacteria, and BCG vaccination [27].

### 4.11. In Vitro Interferon-*γ* (IFN-*γ*) Release Assays (IGRAs)

A rapid and accurate tool for diagnosing childhood TB would be highly beneficial. Much attention has been focused on immune-based assays that do not rely on sputum but can be done with blood [31]. This assay is based on the principle that T-cells of individuals infected with *M. tuberculosis *release IFN-*γ* when they re-encounter TB-specific antigens [3]. IGRAs have been developed to replace the tuberculin skin test (TST) for detection of latent TB infection (LTBI) [19, 31]. Latest versions of IFN-*γ* assays use antigens such as the early secreted antigenic target 6 (ESAT-6) and culture filtrate protein 10 (CFP-10) [26]. These antigens, encoded within the region of difference 1 (RD1) of the *M. tuberculosis *genome, are significantly more specific to *M. tuberculosis *than PPD, as they are not shared with any BCG vaccine strains or selected NTM species including *M. avium* [3, 31]. The commercial tests with this fundament are Quantiferon TB-gold (QFT-G) and enzyme-linked immunospot assay (ELISPOT). They are proposed to offer improved specificity over the tuberculin skin test (TST) [4]. 

Dogra et al. in 2007, conducted a study in India in which he compared the results between the TB-gold Quantiferon and TST which obtained comparable results even in malnourished children [4]. Furthermore, Nicol et al. in 2005, reported the specificity between the PPD and the ELISPOT, they found that ESAT-6- or CFP10-specific IFN-*γ*-producing lymphocytes were detectable at diagnosis in two thirds of children presenting with a clinical diagnosis of tuberculosis; however, responses were more frequently positive in patients with culture-proven disease. Due to this, ELISPOT is a promising tool for the clinical evaluation of childhood tuberculosis [3]. Detjen et al. examined the diagnostic value of both commercially available IGRAs, compared with the TST, in the diagnosis of TB. The specificity for TB of QFT-IT was 100% and the specificity of T-SPOT was 98%, both of which were considerably higher than the specificity of TST. Both IGRAs showed high diagnostic value in bacteriologically confirmed childhood TB. Their advantage in this study, when performed in addition to the TST was the ability to distinguish positive TST results caused by nontuberculous mycobacterial disease, thereby reducing overdiagnosis of TB and guiding clinical management [26]. 

Bakir et al. in 2008, reported the prognostic value of IFN-*γ* as biomarker in children and positive ELISpot results predict subsequent development of active tuberculosis in recent tuberculosis contacts, the authors conclude that ELISpot testing could allow more focused targeting of preventive therapy to fewer contacts [39]. However, Nicol et al. in 2009, made a comparison between ELISPOT and the tuberculin skin test in which some children with active TB resulting in negative T-SPOT-TB. These suggest that the test may have impaired sensitivity for very young children, for whom it should not be used to exclude the possibility of active tuberculosis [9]. Moreover, significantly lower production of IFN-*γ* in response to the positive control mitogen phytohaemagglutinin was observed in children younger than 4 years old compared with those 4–15 years old children (*P* < 0.0001) using an in-house ELISA [38].

## 5. New Approaches in TB Diagnostics

### 5.1. GeneXpert MTB/RIF System

GeneXpert includes the development of integrated DNA extraction and amplification systems. This requires minimal manipulation of sample and operator training. It utilizes real-time PCR (rt-PCR) technology to both diagnose TB and detect rifampicin resistance. The test amplifies a region of the *rpoB* gene of *M. tuberculosis*. Mutations of this region give rise to 95% of rifampicin resistance. Resistant strains contain mutations localized within the 81 bp core region of the bacterial RNA polymerase *rpoB *gene, which encodes the active site of the enzyme. In addition, the *rpoB* core region is flanked by *Mycobacterium tuberculosis*-specific DNA sequences. Thus, it is possible to test for *M. tuberculosis *and for rifampicin resistance simultaneously. The simplicity for the user makes this an assay that could feasibly be widely implemented outside centralized laboratories and potentially impacts on TB control [47]. The Xpert system has some advantages over the cultivation, mainly in specificity and a shorter time to get results [24].

Recently, Nicol et al. in 2011, reported the application of this method in 452 hospitalized children from South Africa, with or without HIV, with a median age of 19.4 months, and suspected of having TB. Two Xpert tests doubled the case detection rate compared with smear microscopy (76% versus 38%), identifying all smear-positive and 61% of smear-negative cases, the specificity was 98.8%. The sensitivities for smear-negative TB were 33.3% and 61.1% when testing one or two samples, respectively. The samplings were induced sputum and they detected three quarters of culture-confirmed tuberculosis with very high specificity; the yield of this method was twice that of smear microscopy. This could suggest the possibility of replacing the microscopy for this type of methodology which has greater sensitivity especially with a second sample [40] (Table 3).

### 5.2. Gas Sensor Array Electronic Nose (Electronic Nose)

The potential to detect different *Mycobacterium* species in the headspaces of cultures and sputum samples is another innovative approach that is currently in development. The array uses 14 sensors to profile a “smell” by assessing the change in each sensor's electrical properties when exposed to a specific odour mixture. In an initial study using sputum samples from patients with culture-confirmed tuberculosis and those without tuberculosis, the E-Nose correctly predicted 89% of culture-positive patients with a specificity of 91% [24]. In a further development applying advanced data extraction and linear discriminant function analysis, obtained sensitivities were of 68% and 75%, and specificities of 75% and 67% for Rob and Walter electronic noses, respectively [24]. Further applications of this test, including its potential value in the diagnosis of child tuberculosis, are needed.

### 5.3. Other Developments in Progress

Recently, it has been produced llama (*Lama glama*) antibodies specific for the 16 kDa Heat Shock Protein of *M. tuberculosis, *but testing in patients samples has not been done yet [48] and a fast method to diagnose active pulmonary TB by infrared spectroscopy of serum blood sample seems promissory [49, 50]. Also, a method to accelerate *M. tuberculosis* growth in culture applying alternating magnetic fields frequencies of 8 Hz and amplitudes of 80 E for 4-5 days was developed by Noreiko et al., reducing by 7-fold the time in getting results when compared with traditional culture [51]. With another relevant development, it is possible to discriminate among lung diseases as cancer, asthma, and TB by gas sensor technology, which monitor exhaled volatile organic compounds directly from breath of patients, and this can be applied to diverse equipment as the above-mentioned E-nose [52].

## 6. Concluding Remarks

Tuberculosis is the leading cause of death worldwide in both adults and children, and the incidence and prevalence in the latter are underestimated due to the difficulty to collect samples and the lack of efficient methods to detect reaction specificity with no cross-infection with other mycobacteria. There are several methods currently being used for TB diagnosis in children, some classical approaches comprise mycobacterial culture, microscopy, TST, IGRAs, and more recently high-tech diagnostics approaches as real-time PCR (GeneXpert), E-nose, and infrared spectroscopy. 

After the above mentioned, we can propose the identification of mycobacteria in children by two different methods. The first would be for use in places where access to infrastructure and technology is very limited, in this type of scenario, the best option would be a rapid test based on lateral flow device to be used in serodiagnosis, that could be implemented on a test strip to confirm or rule out the presence of bacilli in the patient. In case of having the necessary infrastructure, the GeneXpert would be the best option because it has shown no cross-reactions and also in studies with children have shown a higher sensitivity and specificity than culture methods, but it is clear that a gold standard for childhood tuberculosis is still needed, because to date is not possible to be sure about the presence or absence of the bacillus with a single test in this widely neglected but relevant epidemiological population. 

Regarding the sample type, to date, in pulmonary TB, the sputum induction would be optimal since it is an approach that does not affect the results (as seen in the capsule as stomach acids can inhibit the growth of mycobacteria) and is painless and minimally invasive (unlike gastrointestinal and nasopharyngeal washings). Specifically, when sampling for extrapulmonary TB, blood would be the best option, however, in patients with systemic TB, mycobacteria have been found also in the urine, and this kind of sample, the least invasive to date (not including breath's patient yet), has been poorly explored in adult TB, and even less has been done in children TB research. 

Recent developments for TB diagnostics as infrared spectroscopy (serum) and gas sensor technology (breath or sputum) seem promissory fields due to they are fast and minimally invasive, but their drawbacks are that they must be validated in diverse populations and improved according to the patient's needs. With the above-mentioned ideas, it is clear that we are starting a 1000-mile walk in childhood tuberculosis, but surely the next generation of TB diagnostics tools holds great promises.

## Figures and Tables

**Table 1:** Clinical similarities and differences between adult and childhood TB with relevancy to successful diagnosis.

Feature	Adults	Children
Typical signs	Radiological features and a positive sputum smear	TB can mimic many common childhood diseases. The clinical symptoms in older children are cough, fever, wheezing, fatigue, and failure to gain weight, and in pediatric children are pulmonary parenchymal disease and intrathoracic adenopathy, lymphadenopathy, and central nervous system involvement

X-rays findings	Classical cavitation in lungs	Enlargement of hilar, mediastinal, or subcarinal lymph nodes and lung parenchymal changes, hilar lymphadenopathy with or without a focal parenchymal lesion

TST	Cross-reaction with BCG vaccination and exposition with other mycobacteria	

Sampling	Easy sputum and blood sampling	Difficulty to expectorate, blood sampling usually painful in pediatric children

Bacillary load	High bacillary load, easy to find the bacillus when technician is skillful	Lower bacillary load and is usually smear negative even with fluorescent dyes

Bacillus growth in culture	High yields of 90–100%	Confirmation by culture rarely exceeds 30–40%

Tropism of *M. tuberculosis *	Commonly localized infection in the lungs	Commonly extrapulmonary, disseminated

**Table 2:** Pros and cons of most common diagnostic tests for childhood TB.

Methodology	Pros	Cons
Symptoms	No need for lab infrastructure, diagnostic value if appropriate risk stratification is applied	This criterion has been approved only in conjunction with the TST and suggestive chest radiography

Traditional chest radiograph	The basic equipment is very common in hospitals and some research centers.	The images are not always clear and the lesions in children are often subjective

Thorax CT scan	Enhanced visualization of small lesions not seen on chest radiograph. X-ray high-resolution computed tomography, it is the most sensitive tool currently available to detect hilar adenopathy and/or early cavitation can be used for follow-up	Costly; requires scanner which is not readily available in many settings

Algorithms	They are very helpful and easy to use in countries with restricted technology	Is not commonly used due to lack of validation, it is based on responses of patients to which scores are given which are thought to be very subjective

*M. tuberculosis* culture	Gold standard for definitive diagnosis of adult TB	Culture usually takes weeks (or four days in accelerated culture), low sensitivity (*˂*50% in gastric aspirate/sputum)

*Smear stain *	Rapid	Very low sensitivity (*˂*50% in gastric aspirate/sputum), difficulty in obtaining sputum samples, and poor performance of smear microscopy

Tuberculin skin test (TST)	Very common and cheap reagent, easy to use and to interpret the results	Inespecific, only indicates infection with a mycobacteria or prior BCG vaccination

Polymerase Chain Reaction (PCR)	This is a rapid, sensitive, specific and affordable method	These tests are not performed correctly in all clinical laboratories. The cost involved, the need for thermocycler (or boiling pots at specific temperature), and scrupulous technique to avoid cross-contamination of specimens preclude the use of PCR techniques in many developing countries

In-house nucleic acid amplification assays	Mean sensitivity of 60%, with a proper technique could be done efficiently	These assays are dependent of operator's skill

Adenosin deaminase	This method does not require sputum, only blood. Very high sensitivity and specificity	The report presents unclear case definition, exclusion of nontuberculous patients, and a relatively small TB patient population (20 with active TB)

Serology and antigen detection	In this method, the sample is blood which is easier to obtain than sputum (in PTB). It is very rapid and does not require specimen from the site of disease	Sensitivity and specificity depend on the antigen used

*In vitro* interferon-gamma (IFN-*γ*) released assays (IGRAs)	These methods can replace TST for detection of latent TB infection. Rapid test versions are inexpensive, and dozens of commercial kits are on the market; high specificity (98–100%)	The test may have impaired sensitivity for very young children, for whom it should not be used to exclude the presence of *M. tuberculosis *

GeneXpert MTB/RIF system	This requires minimal manipulation of sample and operator training. It utilizes real-time PCR technology to both diagnose TB and detect rifampicin resistance. Results in ~105 min.	Only one report in a children population from South Africa. There is a need to validate in other populations

Gas sensor array electronic nose (E-Nose)	High specificity	Without data in children populations

**Table 3:** Sensitivity and specificity of commercial and in-house methods for TB diagnostics.

Methodology	Sensitivity	Specificity
Commercial tests		
AMTD Standard (*n* = 15)	0.79	0.91
Smear positive (*n* = 3)	0.98	0.55
Smear negative (*n* = 3)	0.75	0.90
Gastric aspirate only (*n* = 1)	0.73	1.00
Cut-off: 71,000 (*n* = 1)	0.83	0.91
Cut-off: 7,300,000 (*n* = 1)	0.90	0.85
Cut-off: 30,000 (*n* = 1)	0.93	0.66
AMTD Enhanced (*n* = 5)	0.89	0.98
Smear positive (*n* = 2)	1.00	0.90
Smear negative (*n* = 2)	0.83	0.98
Low suspicion of TB (*n* = 1)	0.83	0.97
Intermediate suspicion of TB (*n* = 1)	0.75	1.00
High suspicion of TB (*n* = 1)	0.88	1.00
Amplicor COBAS (*n* = 10)	0.72	0.99
Low pretest probability (*n* = 1)	0.33	0.99
Intermediate pretest probability (*n* = 1)	0.33	0.98
High pretest probability (*n* = 1)	0.47	1.00
Smear positive (*n* = 2)	0.91	0.50
Smear negative (*n* = 3)	0.75	0.99
Amplicor manual (*n* = 20)	0.68	0.94
Smear positive (*n* = 6)	0.91	0.74
Smear negative (*n* = 6)	0.57	0.90
LCx assay (*n* = 5)	0.90	0.96
Smear positive (*n* = 2)	0.98	0.10
Smear negative (*n* = 2)	0.90	0.96
Amplicis Myco B (*n* = 1)	0.92	0.85
Sputum only (*n* = 1)	0.91	0.90
GeneXpert adult population (*n* = 1)	0.95	1.00
Tanzanian adult population (sputum and smear positive, (*n* = 1)	0.88	0.99
Children population		
Two induced sputum samples (*n* = 1)	0.76	0.99
Smear positive (*n* = 1)	1.00	0.99
Smear negative (*n* = 1)	0.61	0.99
In house tests		
IS 986 (*n* = 2)	0.90	0.95
Smear positive (*n* = 2)	0.97	0.83
Smear negative (*n* = 2)	0.75	0.47
Sputum only (*n* = 1)	1.00	1.00
IS 6110 (*n* = 26)	0.79	0.84
Chemical DNA extraction (*n* = 1)	0.60	0.92
Simple boiling (*n* = 1)	0.85	0.98
Smear negative (*n* = 4)	0.90	0.92
Smear positive (*n* = 7)	0.92	0.42
Bronchiestasis only (*n* = 1)	NA	0.86
Upper lobe infiltrates (*n* = 1)	0.67	1.00
Agarose gel electrophoresis (*n* = 1)	0.90	1.00
Dot blot hybridisation (*n* = 1)	0.92	0.98
ELISA (*n* = 1)	0.90	1.00
MTP40 (*n* = 1)	0.97	0.86
MTP40 and *α* antigen (*n* = 1)	0.74	1.00
MPB70 (*n* = 1)	0.98	0.50
Smear positive (*n* = 1)	0.99	0.13
Smear negative (*n* = 1)	0.96	0.53
2.4 kb DNA (*n* = 2)	0.55	0.94
65 kDa (*n* = 5)	0.84	0.85
Gastric aspirate only (*n* = 1)	1.00	0.80
Sputum only (*n* = 2)	1.00	0.84
MPB64 (*n* = 2)	0.56	0.84
2.4 kb DNA and MBP64 (*n* = 1)	0.98	0.70
2.4 kb DNA and MBP64 and 65 kDa (*n* = 1)	0.98	0.70
MTB 10 and MTB 11 (*n* = 1)	0.94	0.94
Ag 85 (*n* = 1)	0.90	0.94
groEL (*n* = 1)	0.82	0.81
Meta-analysis for tuberculous pleuritis	0.92	0.90
ADA (*n* = 63)	0.80	0.84
Pab (*n* = 1)		
E-nose		
Culture positive (*n* = 1)	0.89	0.91
EN Rob (*n* = 1)	0.68	0.75
EN Walter (*n* = 1)	0.75	0.67

Values were obtained from the average of different studies [40–42]. Most data of sensitivity and specificity has not been validated for its application in children populations. AMTD: amplified *Mycobacterium tuberculosis* Direct test, LCx: ligase chain reaction, *n*: number of conducted studies on each test, NA: not available, Pab: protein antigen b.
